# Economically Viable Components from Jerusalem Artichoke (*Helianthus tuberosus* L.) in a Biorefinery Concept

**DOI:** 10.3390/ijms16048997

**Published:** 2015-04-22

**Authors:** Eva Johansson, Thomas Prade, Irini Angelidaki, Sven-Erik Svensson, William R. Newson, Ingólfur Bragi Gunnarsson, Helena Persson Hovmalm

**Affiliations:** 1 Department of Plant Breeding, the Swedish University of Agricultural Sciences, Box 101, SE-230 53 Alnarp, Sweden; E-Mails: Bill.newson@slu.se (W.R.N.); Helena.persson@slu.se (H.P.H.); 2 Environmental and Energy Systems Studies, Lund University, Box 118, SE-221 00 Lund, Sweden; E-Mail: Thomas.prade@miljo.lth.se; 3 Department of Environmental Engineering, Technical University of Denmark, DK-2800 Kgs Lyngby, Denmark; E-Mails: iria@env.dtu.dk (I.A.); inbg@env.dtu.dk (I.B.G.); 4 Department of Biosystems and Technology, the Swedish University of Agricultural Sciences, Box 103, SE-230 53 Alnarp, Sweden; E-Mail: sven-erik.svensson@slu.se

**Keywords:** biogas, leaves, proteins, sesquiterpene lactones, succinic acid, tubers

## Abstract

Biorefinery applications are receiving growing interest due to climatic and waste disposal issues and lack of petroleum resources. Jerusalem artichoke (*Helianthus tuberosus* L.) is suitable for biorefinery applications due to high biomass production and limited cultivation requirements. This paper focuses on the potential of Jerusalem artichoke as a biorefinery crop and the most viable products in such a case. The carbohydrates in the tubers were found to have potential for production of platform chemicals, e.g., succinic acid. However, economic analysis showed that production of platform chemicals as a single product was too expensive to be competitive with petrochemically produced sugars. Therefore, production of several products from the same crop is a must. Additional products are protein based ones from tubers and leaves and biogas from residues, although both are of low value and amount. High bioactive activity was found in the young leaves of the crop, and the sesquiterpene lactones are of specific interest, as other compounds from this group have shown inhibitory effects on several human diseases. Thus, future focus should be on understanding the usefulness of small molecules, to develop methods for their extraction and purification and to further develop sustainable and viable methods for the production of platform chemicals.

## 1. Introduction—Characteristics of the Jerusalem Artichoke for Potential Biorefinery or Multipurpose Use

A concept of rising interest for society is the development of biorefineries. A biorefinery is an analogue to today’s petroleum-based refineries with the difference that the biorefinery is built on renewable biomass resources instead of the petroleum that is the feedstock for today’s refineries. The main reason for the upcoming biorefineries is a wish to transfer from today’s system of fossil fuel use, which is non-sustainable with rising prices in the long term due to depletion of resources [1,2]. At present, a large share of the energy carriers worldwide, as well as materials and chemicals produced, have their origin in fossil resources [1]. Thus, a sustainable society with economic growth and development requires novel solutions based on sustainable use of biological raw material, mitigating climate change and taking development, production and economy into consideration [1,3]. In a biorefinery there is in principal an opportunity to convert almost any type of biomass into almost any type of biofuel, biochemical or biomaterial, if only suitable biotechnological and chemical techniques are combined [1,3]. However, while considering biorefineries, not only production must be discussed but also how sustainable that production is from economic, resource use and social perspectives [3,4].

From the resource sustainability perspective, selection of biomass to be used in the biorefinery is an important aspect. Most literature related to biorefinery research is based on forest biomass, algae biomass, agricultural and/or food waste and crops cultivated on marginal land resources. Literature on crops to be cultivated for biorefinery use is scarce, most likely due to the ongoing debate related to agricultural land to be used for food or fuel production in a world which still sees starvation and malnutrition for part of its population [5,6]. Jerusalem artichoke has some interesting features which make it interesting as a biorefinery crop; it is resistant to most pests and diseases, it is frost and drought tolerant, it can grow on most soils and has low fertilizer requirements [7,8,9,10]. Thus, Jerusalem artichoke can grow on soils were many other food crops cannot grow, and it can be grown further north than many other food crops while still having the potential to yield well (5 Mg/ha dry weight of tubers; 58°20–40'N, Southern Norway) [11]. A recent study also showed that Jerusalem artichoke has the potential for higher dry matter yield (4–35 Mg/ha) from crop residues compared to other crop residues such as corn stover, rice straw, sugarcane bagasse, wheat straw and hemp stem (2–11 Mg/ha; 55°39'N, Southern Sweden) [12]. The high variation in dry matter yield reported (from 4 to 35 Mg/ha) was due to three harvest occasions over the season and 11 clones of Jerusalem artichoke evaluated, meaning that the selection of Jerusalem artichoke clone and harvest time is of utmost importance for high yield. In a study using one clone of Jerusalem artichoke harvested in early autumn, the crop was not found to have similar high potential when compared to a number of other vegetable biomass feed-stocks [13]. As in most research based field trials, the results comparing Jerusalem artichokes clones and harvest dates originated from small hand-harvested plots, and thus the results are not fully comparable to commercial field production of Jerusalem artichoke. Beside the crop residue dry matter yield, Jerusalem artichoke produces tubers with a dry matter yield of 0.45–15.8 Mg/ha (calculations from raw data of Gunnarsson *et al.* 2014 [12]). Data from China reports dry weight tuber yield of 9.1–10.6 Mg/ha and aerial biomass dry matter yield of 18.1–31.3 Mg/ha [14]. Comparatively, grain production of cereals reaches 0.5–12 Mg/ha [15]. The combination of the hardiness of the crop and the high dry matter yield makes Jerusalem artichoke of specific interest as a biorefinery crop.

The most envisioned approach at the moment when it comes to biorefineries is that they should focus on producing chemicals by depolymerization and/or fermentation of biopolymers [16]. One important aspect if the biorefinery is to become a competitive process is that it should produce at least one product of high value (such as a high value chemical or material). Beside chemicals, one energy product should also be produced [1]. Of specific interest are small bioactive molecules that are of use as dietary components in food, as flavors, fragrances, sweeteners, as natural pesticides and as pharmaceuticals [17]. Jerusalem artichoke is known to contain an interesting polysaccharide in its tubers, inulin, amounting to 10%–20% of fresh tuber weight [18,19,20,21], being a dietary fiber and also known to have prebiotic effects [22,23]. However, at present root chicory (*Cichorium intybes* L.) is the main crop for inulin production [24]. Jerusalem artichoke is also known to contain other high value chemicals and small bioactive components of bioeconomic interest if the crop is utilized in a biorefinery concept [9]. The fact that the crop, besides being hardy and high yielding, also contains inulin makes it relevant for further evaluation as a potential biorefinery crop. However, economic and environmental evaluations of the potentials of various components of Jerusalem artichoke have been limited.

The present paper reviews data on Jerusalem artichoke. Additionally, results on variation in protein content in leaves and tubers, and antioxidant capacity in leaves of Jerusalem artichoke between clones and harvest times not previously published are presented. From the review data as well as the new additional data, potential products are discussed. Furthermore, preliminary economic evaluations in the present paper are shown as a concept to reveal the options for Jerusalem artichoke as a potential biorefinery crop.

## 2. Carbohydrates—Types, Content and Potential Uses

Jerusalem artichoke tubers primarily contain two types of carbohydrates, inulin and sugars (fructose and glucose) [12,25]. The main carbohydrates in the aerial biomass are cellulose and hemicellulose [12].

Inulin is an interesting compound from a biorefinery point of view, being a functional food ingredient [23,26]. It contributes to the organoleptic characteristics of food, improves stability of foams and emulsions, and when used as a gel in water it has fat like characteristics [26]. Inulin is degraded to oligofructose through hydrolysis by inulinase [27]. Inulin and oligofructose have been shown to stimulate the immune systems in the body, increase absorption of calcium, and decrease triglycerides and fatty acids content in blood serum; they modulate hormonal levels of insulin and glucagon and reduce the incidence of colon cancer [23]. Oligofructose has technological properties closely related to sugar and glucose syrup [26]. Yield of inulin in tubers of Jerusalem artichoke has been reported to vary between 0.36–12.6 Mg/ha (75.8–84.3 g/100 g dry weight) over the season and in different clones [12].

Inulin and oligofructose are commonly found in nature, being present in around 15% of all flowering plants [23,26]. However, at present there are mainly two species, Jerusalem artichoke and chicory, which are used by industry for the production of inulin [23]. Chicory dry matter yield has been reported of 5.6–7.8 Mg/ha [28] with an inulin content of 70%–80% [23]. Thus, the inulin yield per ha is often higher in Jerusalem artichoke than in chicory. One important aspect for the quality of the inulin is its degree of polymerization (DP). In general the DP was found higher (around 14) earlier in the season than later in Jerusalem artichoke [12]. DP of around 10–12 has been reported for standard inulin from chicory and Jerusalem artichoke, although high performance inulin with a DP of 25 has also been produced from chicory [12,26].

The sugar content of Jerusalem artichoke tubers has been reported to be around 4%–5% of the dry weight [12]. Tubers of Jerusalem artichoke have been evaluated both as substrate for ethanol and succinic acid production with yields of 48% [29,30]. Additionally, l-lactic acid, acetone-butanol, 2,3-butandiol, butyric acid, sorbitol and biodiesel are other products that have been obtained through fermentation processes of the tubers of Jerusalem artichoke [31].

In the aerial parts of Jerusalem artichoke a dry matter cellulose yield of up to 8.8 Mg/ha and a dry matter hemicellulose yield of up to 4.6 Mg/ha have been reported [12], making this part of the crop competitive with other cellulose rich crop residues (corn stover, rice or wheat straw, sugarcane bagasse or hemp stem having maximum dry matter yields of 6.8 Mg/ha for cellulose and 3 Mg/ha for hemicellulose) [12]. However, the relatively low content (measured as % dry weight) of cellulose (11.3–30.8 g/100 g dry weight) and hemicellulose (9.0–17.3 g/100 g dry weight) in the Jerusalem artichoke stalks, the chemical complexity of these compounds and also the high content of lignin in the stalks have limited their usefulness [32]. Recent studies indicate the possibility of using the whole plant of Jerusalem artichoke for ethanol [33] or 2,3-butanediol production [31]. An ethanol yield of 1800–3100 kg/ha from the whole plant has been reported [13,34].

## 3. Proteins—Types, Content and Potential Uses

Protein content of 5.3%–10.4% (dry wt.) has been reported for Jerusalem artichoke tubers, while the aerial parts were reported to have a protein content of 1.1%–6.1% (dry wt.) [12]. While dividing the aerial part of the Jerusalem artichokes into different fractions (leaves, stalk, stump), and analyzing the protein content through the use of the Dumas method on a Flash 2000 NC Analyzer (N conversion factor 6.25 applied) [12,35], a low protein content was generally found in the stalk (1.6%–4.5% with the lowest values at late harvest) and stump (1.6%–2.6% with the lowest values at late harvest). Content of protein was found to be much higher in the leaves of the Jerusalem artichoke (7.1%–24.5%, also with lowest values at late harvest; Table 1). Over all of the seasons and in all different plant parts, the highest protein content was found in the leaves early during the season, with over 20% (dry wt.) protein in some of the clones (Table 1).

**Table 1:** Mean protein content (% of dry matter) measured by the Dumas method on a Flash 2000 NC Analyzer and applying a nitrogen conversion factor of 6.25 [35] in leaves and tubers of 11 different clones of Jerusalem artichoke harvested at three different occasions during the season. For description of the plant material see ref. [12].

Clone	First Harvest (9 September 2011)						Second Harvest (14 October 2011)						Third Harvest (7 December 2011)					
	Leaves			Tubers			Leaves			Tubers			Leaves			Tubers		
1	18.6	±	0.13	6.19	±	0,09	16.6	±	0.04	8.56			8.75			9.28	±	1.90
2	22.4	±	0.04	6.75	±	0.09	16.2	±	1.77	6.75	±	0.00	8.06	±	2.12	8.00	±	1.32
3	23.7	±	0.27	8.31	±	0.09	21.3	±	0.40	5.91	±	0.04	11.3	±	5.70	6.47	±	0.75
4	16.6	±	0.09	8.50	±	0.00	20.8	±	0.84	8.69	±	0.18	7.19	±	2.65	7.19	±	2.48
5	16.3	±	0.13	8.44	±	0.00	8.75	±	0.18	6.75	±	0.27	n.d.			6.69	±	0.80
6	16.2	±	0.18	7.88	±	0.27	9.84	±	0.57	5.25	±	0.00	7.94	±	2.83	7.12	±	3.01
7	19.2	±	0.31	9.38	±	0.53	17.0	±	0.35	5.91	±	0.04	7.50	±	2.03	5.34	±	0.22
8	24.5	±	0.40	n.d.			21.3	±	1.15	7.03	±	0.13	7.12	±	2.21	6.78	±	1.02
9	18.3	±	0.04	n.d.			10.5	±	0.22	6.62	±	0.09	10.3	±	4.42	7.18	±	0.62
10	16.9	±	0.09	7.44	±	0.09	16.4	±	0.44	6.94			9.25	±	4.33	6.06	±	0.09
11	18.3	±	0.00	7.31	±	0.00	16.6	±	1.50	8.06	±	0.09	7.94	±	5.04	6.47	±	0.84

Numbers are representing Mean value ± standard deviation of 2 separate measurements (*n* = 2). When standard deviations are missing, only one measurement was successful. n.d. = not determined.

Limited information is available as to the protein composition in Jerusalem artichoke tubers. In general, the content of amino acids essential for humans is relatively high in Jerusalem artichoke, e.g., higher than in chicory and potatoes. Jerusalem artichoke tubers were also especially rich in sulfur containing amino acids, e.g., four times higher than chicory and potatoes [36]. The combination of Jerusalem artichoke tubers being rich both in essential amino acids and sulfur containing amino acids makes the proteins of this crop of some interest, to be evaluated both for food industry application and as an alternative for the plastics/materials industry. Nutritive value of proteins is always of relevance for the food industry. The content of sulfur containing amino acids may indicate that the proteins have good foaming ability (of importance for food industry but also for e.g., production of insulation materials) [37]. The proteins may also have the ability to form films of good properties as sulfur containing amino acids are related to the formation of disulphide bonds thus building polymeric proteins [38,39,40,41,42]. Besides the option to use the proteins directly in the food or materials industry, proteins are also an interesting source for production of platform chemicals through a first step of degrading the proteins to amino acids from which chemicals can be built [43].

The protein composition of the aerial parts of Jerusalem artichoke has also received limited attention. Two recent studies have focused on developing suitable protocols and methods for proteomic studies of the proteins of Jerusalem artichoke aerial parts [44,45]. These studies report high levels of rubisco in the leaves in Jerusalem artichoke. Rubisco is probably the most abundant protein on earth and makes up between 4% and 28% of the protein in green leaves [46]. Rubisco has a good nutritional profile, comparing favourably with eggs or meat [47,48]. In its purified form spinach rubisco has attractive functional properties with low thermal gelation temperature (approx. 75–85 °C) and relatively low gelation concentration (4% *vs.* 10% for whey proteins) with good foam formation, suggesting use as a replacement for dairy based foams [49]. The ability of alfalfa rubisco to form emulsions can be better than egg white, but its activity depends on the processing parameters [50]. Proteins from the aerial parts may also be further valorised utilizing their ability to form films. Plant proteins have previously been processed into biobased and biodegradable plastics using commercial plastic processing techniques [51,52], to our knowledge rubisco has not been investigated for these non-food applications.

## 4. Bioactive Compounds—Type, Content and Potential Uses

Mean antioxidant capacity of eleven different Jerusalem artichoke clones was found to be 10.2 mmol/100 g DW (mean values ranges 6.6–11.9 among clones) for the tubers and 41.1 mmol/100 g DW (mean value ranges 36.8–47.2 among clones) for the leaves at early harvest (Table 2) by the use of FRAP (ferric reducing ability of plasma) [53,54,55]. The antioxidant capacity in both leaves and tubers thereafter decreased steadily over the harvest season, resulting in values of around 1 mmol/100 g DW in the leaves at late harvest. The FRAP values found for the Jerusalem artichoke leaves at the first harvest (Table 2) are well in accordance with what is reported in many fruits and berries, while those for tubers are lower. Actually, FRAP values found in Jerusalem artichoke leaves at the first harvest are higher than those reported for apple peel (25.2 mmol/100 g DW), and the berries of cranberry, lingonberry, raspberry, sea buckthorn and strawberry (13.9–36.0 mmol/100 g DW) [56]. On the other hand, higher FRAP values have been reported in some berries—bilberry, black currant, elderberry, purple chokeberry, rose hips and sloe (42.1–178.5 mmol/100 g DW) [56]—than in the Jerusalem artichoke green leaves. Berries are well known as sources of bioactive compounds, and the content of phenolic compounds, especially, have been related to human health [57]. Rose hips in particular have been reported in several studies as a rich source of bioactive compounds [58,59] and having high FRAP levels [59]. Vegetables and green leaves, also known to contain bioactive compounds, are normally reported to have lower FRAP activity levels than the berries, e.g., peppers were reported as having the highest FRAP levels among a number of vegetables with values of 15–19 mmol/100 g DW) [60]. For the leaves of Jerusalem artichokes, FRAP values were decreasing over the season and at a second harvest, mean levels had fallen to 23.0 mmol/100 g DW (Table 2). Thus, time of harvest of vegetables and green leaves might play a role for previously reported FRAP values from other sources.

Since ancient times, Jerusalem artichoke has been known in folk medicine as being beneficial for the treatment of diseases including diabetes and rheumatism [61,62]. Bioactive compounds known to be present in Jerusalem artichoke are coumarins [63], polyacetylenes and their derivatives [62,64,65], and sesquiterpenes [66]. Aerial parts of Jerusalem artichoke have shown antimicrobial and antifungal activities [9,67]. Furthermore, recent studies have shown that germacrane sesquiterpene lactones from Jerusalem artichoke have anticancer properties and that these compounds are cytotoxic agents [67,68,69]. Sesquiterpene lactones are compounds known to exert a variety of biological activities, including anti-tumour, anti-inflammatory, cytotoxic, and anti-microbial effects [70]. Of specific recent interest has been the discovery of artemisinin, which is found in *Artemisia annua*, and has gained much popularity as an antimalarial drug, as resistance to other drugs has been growing. Several sesquiterpene lactones are also in clinical tests as anti-cancer drugs and for the prevention of cardiovascular diseases [71].

**Table 2:** Mean antioxidant capacity (mmol/100 g DW) measured by FRAP (ferric reducing ability of plasma) [53,54,55] measured in leaves and tubers of 11 different clones of Jerusalem artichoke harvested at three different occasions during the season. For description of the plant material see ref. [12].

Clone	First Harvest (9 September 2011)						Second Harvest (14 October 2011)						Third Harvest (7 December 2011)					
	Leaves			Tubers			Leaves			Tubers			Leaves			Tubers		
1	44.0	±	0.96	10.8	±	0.65	28.3			5.30	±	0.11	0.60	±	0.03	1.63	±	0.20
2	39.0	±	0.99	8.16	±	0.37	15.9	±	1.38	8.14	±	0.43	0.95	±	0.05	3.57	±	0.24
3	41.6	±	2.12	11.8	±	0.80	n.d.			5.85	±	0.18	1.04	±	0.03	1.25	±	0.05
4	37.6	±	4.27	11.6	±	1.30	17.4	±	0.12	8.94	±	0.18	1.99	±	0.06	n.d.		
5	37.8	±	2.61	7.79	±	1.70	12.7	±	0.96	6.14	±	3.68	0.43	±	0.05	n.d.		
6	36.8	±	4.79	11.5	±	0.61	38.6	±	0.98	10.0	±	0.48	0.51	±	0.16	3.34		
7	42.9	±	2.44	10.6	±	1.20	37.5	±	0.94	9.91	±	0.60	1.13	±	0.02	2.92		
8	47.2	±	1.36	10.8	±	0.51	14.9	±	0.91	9.12	±	0.10	1.17	±	0.04	2.91		
9	43.4	±	2.60	9.99	±	0.70	22.5	±	1.62	5.31	±	0.11	n.d.			3.19		
10	39.2	±	1.76	6.55	±	0.24	19.3	±	0.52	5.35	±	0.31	0.58	±	0.04	2.23		
11	42.6	±	3.06	11.9	±	0.67	22.8	±	0.69	9.51	±	0.01	2.36	±	0.04	2.02		

Numbers are representing Mean value ± standard deviation of 3 separate extractions (*n* = 3). When standard deviations are missing, all three extractions were not successful. n.d. = not determined.

## 5. Economic Aspects of Jerusalem Artichoke Cultivation as a Biorefinery Crop

As mentioned above, a biorefinery should focus on at least one high value chemical or material and one energy product [1]. Thus, for a crop to be a biorefinery/green chemical crop, a similar requirement persists. Besides that, the yield of each of the components is important for positive economics in cultivating the crop. As seen from above, Jerusalem artichoke fulfills many of these requirements making it relevant as a biorefinery/green chemicals crop.

The highest value products do often come from small molecules, such as bioactive compounds that can be used as dietary components in food, as flavors, fragrances, sweeteners, natural pesticides and pharmaceuticals [17]. Jerusalem artichoke leaves show extremely high levels of antioxidant activity (Table 2), higher than has been reported in other vegetables or green leaves [60] and the levels are instead similar to many berries [56]. Thus, for economic purposes, harvests of bioactive compounds from the leaves should be taken into consideration while growing Jerusalem artichoke as a biorefinery/green chemical crop. Highest levels of antioxidant activities were found early during the season (September harvest, Sweden). Also, significant differences were found among the different clones investigated, with the highest levels occurring in clone 8 among the clones we investigated (Table 2). Therefore, if Jerusalem artichoke should be grown as a biorefinery/green chemicals crop, where the highest value product is one or several bioactive compounds, determination of harvest date and clone to be cultivated are important parameters. One type of bioactive compound known to be present in Jerusalem artichoke is sesquiterpene lactones. Sesquiterpene lactones from other sources are used as malaria medications and are in clinical tests as anti-cancer and anti-cardiovascular drugs. It might therefore be possible that the sesquiterpene lactones in Jerusalem artichoke are also the highest value products that can be produced from the crop, although neither commercial processes, nor extraction or purification have been developed, making economics difficult to define.

Proteins, both the rubisco proteins from the leaves and the proteins of less known type from the tubers, are most likely high value products that can be sequentially extracted with the bioactive compounds from the crop. Recent research on lucerne has shown the opportunity to extract rubisco proteins from leaves at the same price as extraction of soy proteins [72,73]. Similar extraction procedures at a similar price are most likely possible for Jerusalem artichoke, which was shown to have a protein concentration in the leaves (20% DW, Table 1) similar to that found in lucerne [72]. Also, the rubisco protein has a better nutritional profile [49] than soy protein and probably better foaming properties [47]. Therefore, rubisco protein should most likely receive a higher price than is obtained for soy protein.

The tubers of Jerusalem artichoke are rich in carbohydrates, while the aerial part also has a high yield per ha, but the % content is somewhat lower with a relatively high lignin content, making aerial parts more difficult to utilize. For the tubers, there are a number of possible uses after the extraction of proteins. The tubers can be utilized for production of inulin, biogas, ethanol or platform chemicals, e.g., succinic acid. Current prices of ethanol are 0.5 USD/kg (1.53 USD/GAL, 1 GAL = 3.79 L, 0.789 g/cm^3^) [74], succinic acid 6–9 USD/kg [75], while the price for natural gas is 3.27 USD/GAL [76] and the price for biogas somewhat lower [77]. Prices for inulin products are around 3–4 USD/kg [78]. From the above numbers it is clearly shown that it is more beneficial to produce platform chemicals, such as succinic acid, with a higher price than the very cheapest ones, e.g., ethanol, if the production costs are relatively similar. Moreover, production of succinic acid is connected with use of CO_2_. Thus an additional environmental advantage in terms of abatement of CO_2_ emissions is achieved. Production of succinic acid by fermentation consumes 1 mol of CO_2_ per 1 mol of succinic acid produced. It has been estimated that CO_2_ emission savings in the range of 4.5–5 Mg per Mg succinic acid produced can be achieved [79].

## 6. Issues Related to the Multipurpose Use of Crops

The multipurpose use of crops with an integrated approach to obtain several products at the same time creates certain demands on the extraction and production procedures [80]. Components need to be properly extracted without interacting negatively on other components that should be extracted or fermented later in the process or with the environment. The sesquiterpene lactone component that has been mostly investigated for extraction and purification purposes is artimisinin, utilized as an anti-malarial drug. Extractions of artimisinin have been carried out using hexane, supercritical carbon dioxide, hydrofluorocarbon HFC-134a, ionic liquids and ethanol [81]. Similar methods can most likely be used for extraction of sesquiterpene lactones from Jerusalem artichoke. However, among these methods hexane is the one that has been most widely used and this method might be the most cost-effective [81]. However, it is also seen as the worst with regard to safety and environmental impact, so if Jerusalem artichoke is to be used as a multipurpose sustainable biorefinery crop, the hexane method might not be the method to be used. Newer and greener methods for extraction of artimisinin are available [81] and might be considered for the extraction of sesquiterpene lactones from Jerusalem artichoke.

As for proteins, extraction methods need to be selected in relation to what proteins are to be extracted. Albumin types of proteins are known to be soluble in water, globulins in salt, prolamins in alcohol and glutelins in acid or base [82]. The proteins in both leaves and tubers of Jerusalem artichoke are most likely primarily of the albumin and globulin types. However, recent studies have shown that it is possible to extract various portions of albumins and globulins from, e.g., Crambe by adjusting extraction and precipitation through various pHs [83].

Recent studies on the production of bio-succinic acid have shown the benefits of removing CO_2_ from biogas and converting it into bio-succinic acid through the use of the bacterial strain *Actinobacillus succinogenes* 130Z [84]. Thus with this system it is beneficial if the same crop can be used as a substrate for biogas and succinic acid as we are suggesting for Jerusalem artichoke. The fact that biogas can be simultaneously upgraded to vehicle fuel by this method as bio-succinic acid is produced increases the economic potential of the use of Jerusalem artichoke for these purposes. Furthermore, recent work has shown that Jerusalem artichoke tubers can be fermented into succinic acid without the use of enzymes, thus the tubers with their high carbohydrate content and relatively simple bioconversion is an attractive biomass feedstock, which also influences the production costs [30].

## 7. Preliminary Economic Analyses of the Use of Jerusalem Artichoke as a Biorefinery Crop

To better understand if and how Jerusalem artichoke can act as a biorefinery crop, we have carried out a preliminary economic analysis on production of various products from Jerusalem artichoke harvested on various occasions. Due to lack of data as to what potential products can be produced from the bioactive compounds of Jerusalem artichoke and production costs/prices of these products, possible small molecule based products are omitted from the analysis. Thus, the economic analysis was carried out on production of rubisco from the aerial biomass, protein and succinic acid from tubers and energy from residues of both aerial biomass and tubers, and based on raw yield data on 11 clones of Jerusalem artichoke harvested at three occasions [12]. Processing efficiencies applied in the calculations have been adopted from the literature (Table 3).

**Table 3:** Assumptions related to biorefinery potential.

Parameter	Unit	Low	High	References
Protein extraction efficiency	[%]	37	80	[85,86]
Rubisco fraction of protein	[%]	4	28	[46]
Rubisco purification efficiency	[%]	80	90	own assumption
Sugar hydrolisation efficiency	[%]	89	95	[30,87]
Succinic acid yield	[%]	67	74	[30]

For calculation of energy yields, an estimation of maximum methane potential has been carried out based on the amount of process residues. Methane production potentials were calculated following previously described methods [88] and literature data for the different compounds (Table 4). Complete degradation of the compounds was assumed. Methane volumes were converted to energy units using the higher heating value for methane of 39.2 MJ/Nm^3^.

**Table 4:** Assumed degradation and methane production potentials in anaerobic digestion.

Parameter	Methane Potential
	[Nm^3^/MgVS]
Residual sugar in tubers ^a^	378
Proteins [89]	516
Lipids [90]	1026
Hemicellulose [91]	430
Cellulose	420
Extractives	400
Uronic acid	292

^a^ Based on the assumption that residues contain only glucose and fructose.

As to the production cost of Jerusalem artichoke for biorefinery purposes, production can be assumed to be similar to that of potatoes for industrial purposes. However, extra costs for more expensive seeds, extra mechanical row cleaning and cost for harvest and transport of the tops needs to be added on the production costs per hectare for Jerusalem artichoke as compared to potatoes. Production costs for potatoes for industrial utilization are around 4000–4400 €/ha [92], and thus Jerusalem artichoke production can be estimated to be 20% more expensive, *i.e.*, around 4800 €/ha. As a sensitivity analysis, the cost range between 3800 and 6000 €/ha was tested. Based on literature data on processing costs (Table 5) an estimation of gross margin for production of succinic acid and biogas from Jerusalem artichoke was calculated according to:

(1) $$Gross\;margin\left[\frac{\text{€}}{ha}\right]=\underset{all\;products}{\sum }\left(Income-Biomass\;poduction\;costs-Processing\;costs\right)$$

**Table 5:** Economic assumptions.

Product	Unit	Processing Costs		Income		References
		Low	High	Low	High	
Methane ^a^	[€/MWh]	41	49	84	87	[93,94]
Protein extraction	[€/Mg]	200	200	5500	11,000	Income data based on market price analyses
Rubisco extraction	[€/Mg]	200	200	16,500	33,000	Income data tripled from mixed protein extract
Succinic acid	[€/Mg]	365	707	912	4561	[95,96]

^a^ Processing costs refer to biogas fermentation and upgrading process and income refers to vehicle fuel.

Processing costs for fermentative succinic acid production were calculated based on previous investigations [95] assuming a sugar conversion efficiency of 91% [96] for profit margins between 10% and 30%.

**Figure 1:**
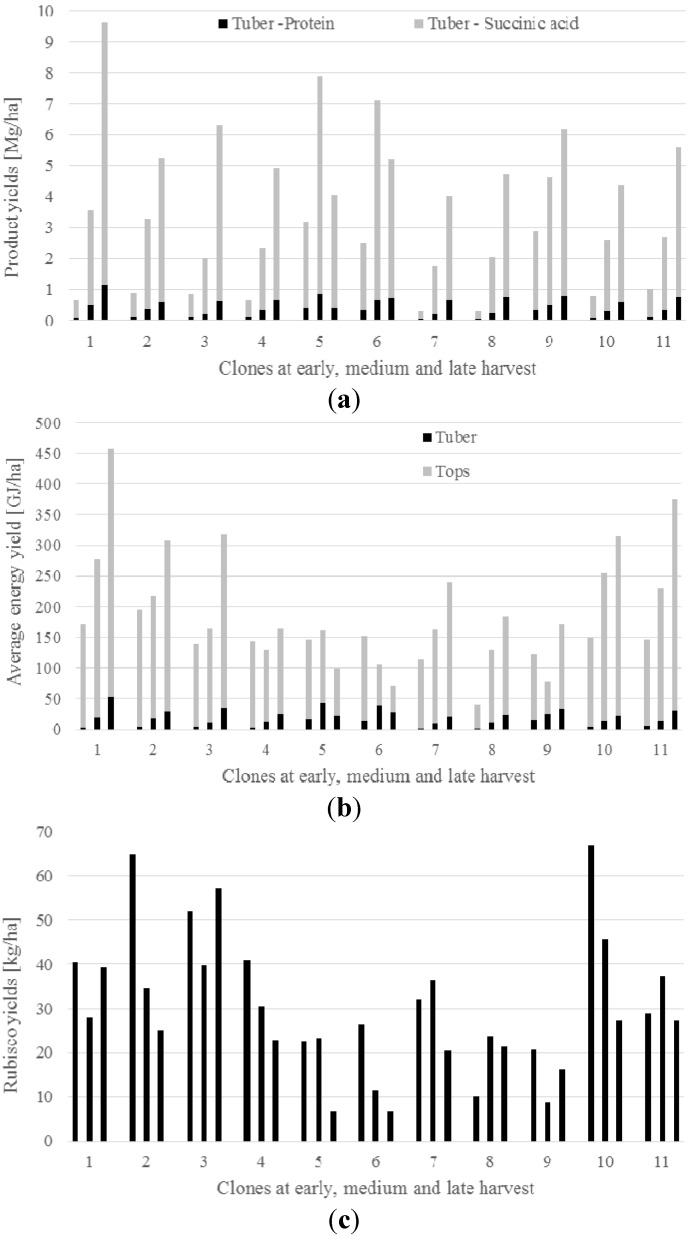
Average yields of (**a**) protein and succinic acid from tubers, (**b**) energy from biogas production of fermentation and extraction residues from tubers and tops, and (**c**) rubisco protein from tops, for different clones of Jerusalem artichoke harvested at early (September), medium (October) and late (December) harvest date.

Based on the mentioned estimations, high yields of succinic acids was obtained from the tubers of all the 11 clones of Jerusalem artichoke with an increasing yield at late harvest dates (Figure 1a). Similarly, protein and energy yields from the tubers as well as energy yields from the aerial parts increased with later harvest dates (Figure 1a,b), although variation among clones was found for all four products. As to rubisco protein yields from the leaves, a decrease was noted with later harvest dates (Figure 1b,c), also here with large variation among clones.

**Figure 2:**
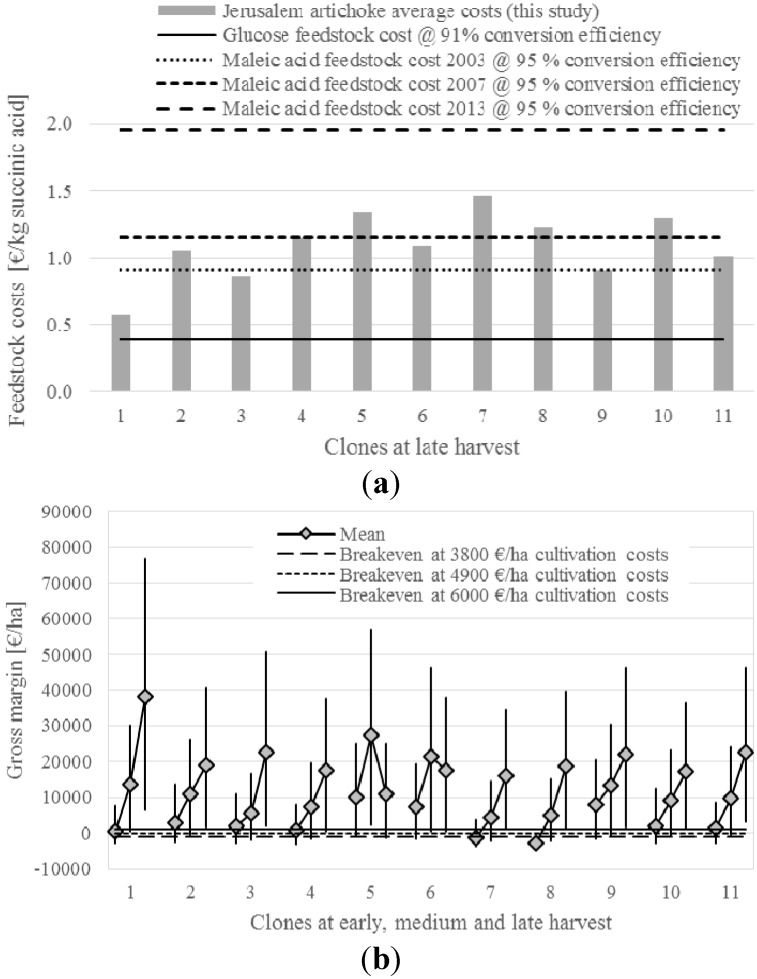
(**a**) Feedstock costs per kilogram succinic acid produced and (**b**) average gross margin of biorefinery utilization at three harvest occasions, of different clones of Jerusalem artichoke. Solid lines in (**a**) represent feedstock costs for glucose as a substrate for bio-based succinic acid fermentation, while the dotted lines show the development of feedstock costs for maleic acid as from petroleum origin [99,100]; In (**b**) grey markers represent average gross margin and black bars represent range according to variation in original chemical analyses and low/high variation for processing efficiencies and costs.

From the present economic analysis, extracted protein from the tubers was the product of Jerusalem artichoke with highest impact on the income, contributing on average 29%, 39% and 45% of the total income at early, medium and late harvest, respectively. Succinic acid from the tubers contributed 28%, 41% and 42% and rubisco from the leaves contributed 21%, 11% and 9%, respectively. Biogas from tubers and aerial biomass process residues contributed 21%, 12% and 10%, respectively. Thus, succinic acid showed the second highest impact on the income. Despite that fact, sole production of succinic acid from Jerusalem artichoke, meaning that this product should bear the full production cost of the crop and of the processing, is hardly competitive compared to the use of other sugar feed-stocks for succinic acid production (Figure 2a). Production costs for production of succinic acid via catalytic hydrogenation of petro-chemically derived maleic acid or maleic anhydride are currently still lower than the succinic acid derived from carbohydrate fermentation [97,98]. However, bio-based succinic acid is becoming more competitive as prices for maleic acid are increasing (Figure 2a) [99,100]. Also, recent studies using Jerusalem artichoke as a feedstock have shown high yields from direct fermentation of the hydrolysis broth [84], and purification costs can likewise be lowered by carrying out subsequent conversions directly in the fermentation broth.

Generally, average gross margins for biorefinery utilization of Jerusalem artichoke were highest at late harvest, with exception of clones 5 and 6, with highest gross margins at medium harvest (Figure 2b). Clone 1 showed an exceptionally high gross margin at late harvest, while results at early and medium harvest were comparable to the other clones. At medium cultivation costs and a 20% profit margin, refining of the clones 1, 4, 7 and 8 became economically viable starting with medium harvest, while the other clones were economically viable at all harvest dates.

## 8. Conclusions—Can Jerusalem Artichoke Be Seen as a Potential Biorefinery Crop?

Jerusalem artichoke can definitely be seen as a potential biorefinery crop. However, a multi-purpose use of the crop for sequential production of several products seems beneficial. For economic profit, the products of highest economical value from the crop have to be defined for a biorefinery utilization of the crop. Potential such high value products are those for medical uses, and bioactive compounds (in this crop probably sesquiterpene lactone mediated ones) from the leaves are specifically interesting. Furthermore, the rubisco protein from the leaves might be of relevance to be used in the food and materials industry. Suitable extraction methods to obtain the bioactive compounds and rubisco proteins in a pure, secure and suitable conformation and to a reasonable price need therefore to be worked out. The wastes after extraction of bioactive compounds and rubisco proteins from the leaves should preferably be used for biogas production, as should the rest of the aerial parts of the Jerusalem artichoke.

The protein of the tubers might be of relevance to be utilized by the food industry and economic analyses indicated the tuber protein as a large part of the economic benefit of the crop. Suitable extraction procedures need to be developed here as well. However, the carbohydrates in the tubers must be seen as the main product of the tubers and an economically viable use of these is currently a necessity to succeed with a biorefinery use of the crop. The carbohydrates of the tubers should preferably be used in a biorefinery concept for succinic acid or other relatively high value platform chemicals production. Eventual residues can be added to the residues of the aerial parts of the Jerusalem artichoke and be utilized for biogas production.

## Definitions

1 € (Euro) = 1.10 US$, 1 ha (hectare) = 10,000 m^2^.

Nm^3^ = normal cubic meter (0 °C, 1.01325 bar (absolute)).
